# The Amelioration of Olfactory Acuity upon Sexual Maturation Might Affect Food Preferences

**DOI:** 10.3390/nu1010003

**Published:** 2009-06-10

**Authors:** Enrico Bignetti, Fiorella Sinesio, Gaetano L. Aiello, Carlo Cannella

**Affiliations:** 1 Lab. “Food Neurochemistry”, University of Parma, 43100 Parma, Italy; 2 “National Research Institute for Food and Nutrition” (INRAN), Viale Ardeatina 546, 00178 Roma, Italy; Email: sinesio@inran.it (F.S.); carlo.cannella@uniroma1.it (C.C.); 3 Dept. of Physics & Related Technologies (DIFTER), University of Palermo, Viale delle Scienze (Ed. #18), 90128 Palermo, Italy; Email: aiello@difter.unipa.it

**Keywords:** olfactory threshold, menstrual cycle, testosterone, trimethylamine, fish consumption, food preferences

## Abstract

Upon sexual maturation, olfactory acuity in women ameliorates and starts oscillating across the cycle. During ovulation, mean olfactory threshold is 30 times lower than during bleeding. Interestingly, menstruated women undergo maleodorant trimethylaminuria. We argued that olfactory amelioration during ovulation might concur to a mating strategy, whereas olfactory impairment during bleeding might protect women against self-refusal. Testosterone and its 17β-estradiol derivative might be responsible for the synchronization of these menstrual events. Furthermore, we posed the question whether olfactory detection amelioration upon sexual maturation might provoke a change in food preferences, for instance a reduction in fish consumption. A preliminary survey in Italy provided encouraging results: 15-44 year-old women have lower fish consumption than 3-14 year-old girls. Surprisingly, men exhibited the same behaviour, so new olfactory tests as well as testosterone measurements are under way.

## 1. Introduction

Volatile chemical signals play a crucial role in human behaviour associated with social interaction, comunication and feeding. As a matter of fact, humans can distinguish a thousand odorants within an extraordinarily vast landscape of synthetic and natural odorants [1].

One major question was whether odors are perceived by women and men in the same way [2,3,4,5,6]. The question is far from being academic, as it may finally provide an answer to the question of whether women and men are equally concerned in the achievement of same social and professional goals. To this regard, we will discuss some cases in which trimethylamine (TMA) - the odor of rotten fish - is implicated in our daily-life situations besides our eating habits. An experiment dealing with the perception of TMA in men and women may reveal gender-dependent differences, with intriguing implications in evolution. In the most trivial case, knowing whether men and women exhibit the same aversion to, or preference for seafood, based on their olfactory discriminants may provide precious insights into the dietary habits of a family, and/or may direct someone to a career in catering.

The sensory modality by which fish is perceived, besides developing an attitude towards this food, may reveal several psychological constructs, such as beliefs, interest, motivation, even social norms, knowledge and other behaviours, all of which may be of interest to the social sciences and food marketing.

As an example, Olsen [7] showed that there is a positive relationship between age and frequency of seafood consumption, which he claimed is essentially mediated by three parameters: the psychological tendency of consumers to eat seafood, including favour-disfavour, liking-disliking attitudes (“A”), health involvement (“H”) and perceived time to prepare meals or convenience (“C”). By means of a questionnaire, he tried to predict the relative weight of the three parameters and the degree of cross-influence among them. In particular, he claimed that sense of smell must be considered relevant for determining “A” but no specific study on fish odor detection or hedonic response was reported. Moreover, it was not taken into account that olfaction can diminish in the elderly (see below). Since this reduction may lessen fish aversion, it may primarily affect the parameter “A” and, indirectly, also “H”. In conclusion, without a specific study, the absolute value and the degree of correlation between these parameters cannot be correctly assessed. This, and other examples, pointed our attention to the need to explore how fish smell is perceived by humans of both sexes, at any stage of their life. 

The following section of this work will critically review the state of the art of sex-dependence in human olfaction, with specific references to the modulation of olfactory detection threshold occurring during the menstrual cycle in women, on one side, and some TMA features, on the other. These argumentations will enable us, in the third section, to pose the crucial question whether olfactory acuity amelioration upon sexual maturation might affect the alimentary habits of women. In the attempt to give an answer, we brought to light a series of surveys on fish consumption in Italy which were carried out by the Italian Ministry of Health. These data could be statistically analysed by us and preliminarly correlated to the olfactory data obtained by others. The correlation between the two sets of data seem to reveal an interesting covariation between olfactory detection thresholds and fish consumption upon sexual maturation. On the basis of this preliminary evidence we propose new tests that might unveil some hidden aspects of this particular sensory modality in relation to a specific hormonal state.

## 2. Sex Differences in Olfaction

The question of whether the human olfactory threshold could depend on sex has been addressed by several authors. Two volatile C-19-steroids, considered putative pheromones in humans, were found [8,9] to contribute to mood, memory and autonomic system responses in a gender-specific manner. A sex-specific difference in olfactory sensitivity for these two molecules was also demonstrated in primates. A clear-cut example of sex-specific bimodal olfactory sensitivity was demonstrated in spider monkeys, with the male responding to only one molecule and the female to the other [6]. However, these molecules carry well-defined physiological information targeted mainly to sex behaviour, with scarce relevance to the mechanism of odorant perception and recognition.

By studying specific anosmia to “primary odorants”, Amoore came to the conclusion that there was a logarithmic deterioration in olfactory perception with age, which was unaffected by sex [2]. In a relatively more recent paper [4], the first easy-to-administer standardized test of human olfactory function was reported. The typical test was based on a “scratch ‘n sniff” method which utilizes 50 different microencapsulated fragrances, trademarked by the 3M Company. The list included common flavours, like many foods and beverages. These fragrances were presented to many subjects in coherent groups and rated according to parameters, such as intensity, pleasantness, familiarity, and so on. Moreover, quite an interesting experiment was reported, which monitored the influence of gender and age over 144 subjects. The results indicate that olfaction declines both in men and women past 60s, with women exhibiting lower odorant thresholds than men across all age groups (from 10 to 90 year old subjects). Pre- or post-pubertal conditions did not furtherly modulate women’s olfaction. 

Later on, several papers tried to disprove sex influence on olfactory threshold. One of them [10] is worth noting, since TMA was used as test-odorant to show there was no correlation between sex and olfactory acuity. Instead, a correlation was found between age and the fish odorant, with the youngest being most sensitive and the adults least sensitive to the odor. The final conclusion was that sensitivity to TMA per se has no significant impact on the subjects’ preferences for, or aversion against fish as food, and therefore the TMA test was deemed of minor importance for assessing fish food acceptability.

The results of experiments on olfaction, however, are always open to debate. One reason is the intrinsic complexity of the olfactory system, and the lack of standardization of methods. An emblematic case is reported by some authors [11]. By using a large sample of women and men, these authors investigated gender-related differences in several tasks aimed to study: absolute sensitivity, intensity discrimination, quality discrimination, episodic recognition memory, and identification. Although a better performance was registered by female subjects on episodic odor memory capability towards familiar odors, no sex related differences in sensory acuity were observed. According to the authors, women’s superiority in episodic odor memory does not depend on olfactory acuity but is largely mediated by their peculiar proficiency in odor identification. 

Collectively, by looking at these experiments, one can always find significant drawbacks in the methodologies and protocols used; like large hormonal dishomogeneity within subjects in the same sample, which may cause a large intraspecific error. If the sample is made up of women, one should also consider the menstrual cycle as a crucial physiological parameter. Infradian hormonal oscillations (corresponding to: menstrual, follicular, ovulatory and luteal or progestagenic phases) are known to profoundly alter a woman’s normal lifestyle. In other words, a different way of perceiving odors during the cycle may affect some habitual choices. Therefore, the takehome message was that any psychophysical measurement of olfactory threshold in women should also ascertain whether the subjects had used oral contraceptives for at least six months prior to test or not.

In a relatively old review on sex differences in human olfaction [12] a series of complex and often contradictory, though interesting examples was reported, that lead the author to conclude with an open enigma. Among all the intriguing references reported, the authors mentioned some cases of olfactory sensitivity variation during the menstrual cycle, in women either taking or not taking the pill. Apparently, these variations were determined by an infradian rhythm possibly activated by the central nervous system and not by specific hormones. 

In our opinion, the examples reported in the literature to date display uncertainty in data collection and interpretation for the following reasons: either the data collection was lacking from a statistical point of view or a bias prompted by an erroneous prediction was causing a wrong data interpretation.

### 2.1. Olfaction and Menstrual Cycle in Women

In a recent paper [5], the authors discussed olfaction gender-dependence in a statistically-convincing manner. They monitored olfactory thresholds of the banana-like odor (amyl acetate) in three different groups of subjects. In group 1 they collected data from a large sample of women (hundreds) at different stages in the menstrual cycle. In group 2, the test was performed repeatedly on many women for the duration of the cycle. Finally, they compared the responses of the women in group 1 at the ovulatory phase, with the responses of pre-pubertal women, post-menopausal women and sexually-mature men (group 3). 

It was evident that olfactory acuity improved upon sexual maturation. In fact, the mean olfactory detection threshold started oscillating across the cycle, between the highest value at menstruation (identical to that of pre-pubertal girls) and the lowest at ovulation (about thirty times lower than at menstruation), with intermediate values at follicular and luteal phases (both three times lower than at menstruation). 

Several explanations were tentatively given by the authors to account for a variation of the thresholds with the phases of the menstrual cycle. None of them, however, was convincing. One was the plasticizing effect of the ovulatory hormones on the olfactory district, similar to that observed on the vagina. This hypothesis was apparently sustained by the observations [13] that receptors of gonadal hormones, particularly estrogens, could be found in the olfactory epithelia. However, since women whether taking the pill or not underwent the same changes in olfactory thresholds across the cycle, the hormonal explanation was not conclusive [14]. Therefore, the menstrual cycle’s influence on the olfactory threshold is either a by-product of other parallel physiological events, or it hides an important biological event whose teleological meaning is still unclear. 

This second hypothesis seems much more appealing. Thus, we discuss the reasons why olfactory sensitivity for the banana-like odor is low during the menstrual phase, with median threshold running about 30 times higher than in the ovulatory phase, and about 10-times lower than in control men. The reason may lie deep inside those very mechanisms that differentiate women from men. A gender dependent olfaction in female rats [15] was shown to be strictly related to sex hormones, with a peak of sensitivity during estrus. In analogy with the findings of Navarrete-Palacios *et al. *[5], this clearly indicates that, somehow, cyclical factors affect olfactory efficiency, although the final explanation of how that link may operate is still lacking. In the attempt to fill this gap, we looked into the complex hormonal circuitry regulating menses, and found some evidence that dopamine metabolism and its regulation may play an important role. 

For many years, researchers have focused their attention on the hormonal and physiological parameters of some aspects of the menstrual cycle in women, like mood swings and behavioural changes. Motor or intellectual skills were unchanged, while weariness, depression, fatigue and irritability were experienced. This is in line with the influence of the autonomic nervous system and cathecolamine metabolism in mood regulation [16]. 

At that time, the hypothesis that the same effectors regulating menses may tune olfactory thresholds via a specific dopamine circuitry was not yet considered. Only very recently was it discovered that intrinsic dopaminergic neurons are abundant in the olfactory bulb, positively modulating odor perception and discrimination through D1 and D2 specific receptors [17,18,19,20]. In our opinion, these novel results may lead to a better understanding of olfactory perception and discrimination mechanisms and may also throw a light on olfactory threshold variations in menstruating women.

For the first time in a vertebrate sample (the female European eel), it was demonstrated that testosterone could be synthesized in the female gonads, secreted into the blood stream, taken up by the olfactory bulbs and, then, partially aromatized into the estrogen 17β-estradiol by a local enzyme [21]. Testosterone and estradiol were supposed to increase dopaminergic tone in the eel’s olfactory bulb.

In humans the effect of androgens and estrogens on the dopaminergic circuitry in the olfactory bulb have not yet been clarified, therefore it is conjecture yet to be evaluated. In fact, there were several indicationsas that peripheric as well central dopaminergic systems were modulated by these hormones. In contrast to fish, these hormones generally exerted a down-regulation of dopamine metabolism and function both in vivo and in vitro. In rat kidneys, for instance, the activity of tyrosine hydroxylase (TH), the rate-limiting enzyme in the dopaminergic synthetic pathway, diminished after a chronic treatment of the animal with testosterone [22]. As regards estrogens, there was evidence that estradiol (and not testosterone) was a crucial down-regulator of dopaminergic tone in the central nervous system [23]. In addition, it was demonstrated in-vitro that estradiol can up-regulate the expression of human progesterone receptor, a member of the nuclear superfamily steroid-receptors [24] thus enhancing cell sensitivity to progesterone.

### 2.2. Trimethylamine: An Odor That Might Unveil the Question of Olfactory Sensitivity Changes in Cycling Women

Even though a hormonal role in olfaction modulation is plausible, the teleological significance of menses synchronized with olfactory threshold fluctuation is still obscure. It may be necessary to approach the issue from the other way around, i.e. by looking at the odors that may recur during menses. These odors may reflect significantly on female physiology and social behavior. Trimethylamine (from the family of interesting odors), emerged as one of the most familiar; i.e. an odor quite frequently encountered in our daily life.

Methylamine, dimethylamine and trimethylamine are derivatives of ammonia that have the typical rotten fish odor at lower concentrations; they have an ammonia-like odor at higher concentrations. Human studies conducted to assess odor perception of these compounds, demonstrated that all three are considered maleodorant, and can be perceived by dissolving them in water at rather high concentrations; TMA being the one with a lower threshold, 3-4 orders of magnitude lower than the others [25]. A significant proportion of human subjects exhibited a pronounced anosmia to volatile tertiary amines (a specific TMA anosmia was monitored in about 7% of them) and to a lesser degree to primary and secondary amines [26]. 

The above substances are present in the decomposition of plants and animals as products of microbial degradation of animal byproducts. As far as fish is concerned, TMA derives from the enzymatic reduction of the non-volatile precursor trimethylamine oxide (TMAO), which is caused by bacteria on the skin [27]. Among the cultures isolated from spoiling fish, *Shewanella putrefaciens* were identified as the major agent responsible for production of TMA and off-odour [28]. Bacterial spoilage and its associated malodor production in fish can cause natural avoidance behaviour in consumers. Thus, controlling these processes was of primarily importance in industrial fish meal plants. New packaging methods and new additives proposed in mitigation strategies, were succesful even at great distances from industrial plants (http://www.freepatentsonline.com/6576281.html). On the other hand, research has tried to further improve ways of detecting fish odors instrumentally, with detection thresholds comparable to the human nose. To this end, a TMA gas sensor was developed and utilized to detect fish freshness [29]. More recently, specific chemical analyses of malodorous compounds downwind of a fish meal were carried out by means of gas chromatography in tandem with mass spectrometry, and the results were compared with human panelist data [30].

TMA is found not only on spoiling fish but also in gastric juices, in the saliva and in the blood of humans and other mammals. It is considered as a normal constituent of mammalian waste, such as feces, urine and exhaled air. The extent of TMA excretion may be influenced by the diet, especially when it contains fish [31]. 

More rarely, the extent of excretion may become abnormal in the case of certain pathological conditions. Striking examples are the cases of “Bacterial Vaginosis” (BV) [32] and of “Primary Trimethylaminuria” (also known as “Fish-odor-syndrome”) [33]. The first commonly affects women of childbearing age, with an occurrence of 10-31 % in various populations, and can be easily assessed on the basis of TMA detection in vaginal secretion. Conversely, the second is a rare invalidating syndrome which involves the exhaling of a rotting-fish odor. Moreover, BV seems to be more elusive than “Fish-odor-syndrome” since the former may be due to concurrent conditions, such as lifestyle, diet, and the microbiologic environment and specific metabolic conditions in vaginal secretions [34,35,36]. Instead, the etiopathogenesis of the “Fish-odor-syndrome” has been elucidated to be a homozigotic genetic defect. This defect is caused by a missense mutation of a gene codifying for the flavine-containing mono-oxigenase 3 (FMO3), the enzyme which converts TMA into a non-odorous metabolite in the liver [37,38]. 

Actually, menstruation can be functionally correlated to transient trimethylaminuria in self-reported subjects suffering from malodor, and also in healthy women with active FMO3. In fact, besides the autosomal recessive defect which produces a totally inactive FMO3, there is also a polimorphic landscape of mild mutations of the FMO3 gene which causes variable degrees of trimethylaminuria during menses. 

The existence of trimethylaminuria during bleeding days in women was discovered long ago [39], but the enzymatic mechanism that regulates odor discharge has been explained only recently by Shimizu *et al. *[38]. According to these authors, a down-regulation of enzyme expression and activity may be observed just around menses in women genotyped for the homozygotic wild FMO3 gene. On the contrary, they observed an up-regulation of FMO3 induction during pregnancy, in accordance with the previous results of others [40]. 

On the basis of this evidence, one may argue that FMO3 gene expression and activity are regulated by the same hormones regulating menses in women. As expected, in a recent paper, some authors [41] demonstrated that testosterone and estradiol exhibited modulatory effects on the various FMO isoforms in Japanese Medaka fish and that this effect manifested itself differently in a clear sex-dependent manner. In particular, FMO3 gene expression in the female liver was strongly down-regulated by circulating testosterone.

### 2.3. The Need for a Model Reconciling Several Physiological Aspects under the Control of Testosterone

Due to a specific FMO3 deficiency during menstruation, women undergo a peak of malodorous trimethylaminuria and a worsening of olfactory sensitivity. By observing the dynamics of these processes, it appeared that these systems were undergoing a common regulatory fate, i.e. as though both systems were under the control of the same hormones regulating menses. Starting from this concomitance, one may infer that olfactory thresholds are tunable just to accomodate women with specific physiological necessities. In this view, a worsening of odor perception during the menstruation may be interpreted just as a self-defence mechanism to avoid the surge of self-disgust in concomitance with trimethylaminuria (and with other possible malodorant excretions in vaginal discharges). 

Conversely, the extraordinary olfaction activated at the ovulatory phase, may be used as a refinement of one of the most sophisticated tools in the search for a mate. In female rodents, for example, the main olfactory system, like the accesory neuroepithelium, contributes to receptivity towards sexually mature conspecific [42]. A specific pathway signalling the detection of volatile male pheromones to medial amigdala thus opening the access to superior diencephalic structures that control mating processes, has been demontrated [43]. When talking of humans, however, we mean not only a ‘simple’ interplay of pheromones, but other refined smell-based strategies, such as the use of agreeable perfume, the choice of the best smelling partner, or the avoidance of other competing women in the proximity etc.

Moreover, a positive FMO3 induction was observed in pregnant women. Maybe this is the response of the mother’s liver to the need for extra detoxifying work required by foetus metabolism. 

As far as young fertile men are concerned, it is interesting to note that their mean olfactory threshold is ten times lower than that of women during menstruation [5]. This may indicate that men are sensitive enough (though unaware of it) to menstrual odors to be able to sexually discriminate women who are not ready to mate. 

In our opinion there may be a hidden functional cross-correlation between phases in the menstrual cycle, liver detoxification and olfactory threshold variation. These physiological processes, though apparently very diverse, pertaining as they do to very different anatomical districts, may actually work under the control of a same hormonal system. At the end of the day, this mechanism may be interpreted as one of the many concurring for a sole result: the selection and propagation of the human species. In this perspective, it should also be possible to account for some ancestral mechanisms of olfactory and hormonal adaptation to a common genetic FMO3 polymorphism. 

**Figure 1:**
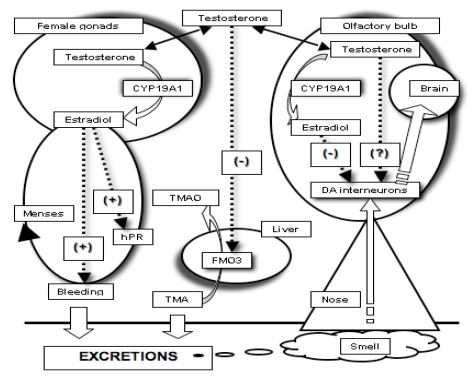
Hypothetical view of the multiple physiological processes concurring to modulate olfaction and liver FMO3 enzyme activity in women during menses. (CYP19A1 = Aromatase enzyme; Estradiol = 17β-Estradiol; TMA = Trimethylamine; TMAO = Trimethylamine oxide; FMO3 = Flavin mono-oxygenase 3; DA interneurons = Dopaminergic neurons; hPR = Progesterone receptor).

Figure 1 shows processes which may be activated during menstruation by assuming that: 1) gonadal testosterone partially undergoes enzyme aromatization to estradiol (whether women are taking the pill or not); 2) then, testosterone may reach different districts; 3) in the vagina, estradiol moderates/modulates bleeding and enhances sensitivity towards progesterone; 4) testosterone down-regulates FMO3 gene expression in the liver; 5) in the olfactory bulb, testosterone may be partially aromatized to 17β-estradiol, so that testosterone and estradiol can modulate the dynamic range of the dopaminergic tone thus tuning olfactory thresholds.

Up to now, the model proposed in Figure 1 has been a synthesis of experiments on women, rats, fish, eels and even *in-vitro* cells; the result is a sort of chimera under the control of testosterone and derivatives. The question is, can we support these assertions with the experimental evidence obtained by monitoring the effects of testosterone on women?

As one can see in Figure 1, we propose a pivotal role of testosterone in synchronizing bleeding, trimethylaminuria and olfactory down-regulation. In syntony with this proposal, testosterone levels in blood increase around mid-cycle [44,45]. This evidence is compatible with our model since down-regulation either of FMO3 gene in liver and olfaction in the olfactory bulb must occur with a reasonable delay with respect to testosterone arousal in blood. 

As far as it regards the lack of pill effect on olfactory modulation during the cycle (see above), one might conclude that the hormonal pathway proposed by us, should by-pass any possible pill interference. However, the menstrual cycle depends on such a complex hormonal interplay that one should be extremely cautious when drawing any conclusion. Taking the pill, maybe it is not a question of olfactory detection imparement but of tuning of preferences. Infact, some authors recently observed a tuning of preferences from social cues with reproductive significance, towards environmental fragrances, like those related to nutrition [46]. 

As far as olfaction is concerned, the use of TMA odor as a probe to reveal whether our model is correct is feasible. Up to now, to test the effect of menses on female olfaction amyl-acetate has been used, a strongly attractive odor. If tests with TMA, one of the most aversive odors excreted with body fluids during menstruation, confirm these results, then a further step has been taken towards understanding the cyclical behaviour of olfaction in women. 

The protocol used by Navarrete-Palacios *et al.* seems the most appropriate, mainly because of the large groups engaged in the tests, the odor used, the sniff-bottle technique, and the procedure of ascending concentrations [5]. Thus, we could adopt the above protocol almost entirely, except for the replacement of the pleasant amyl acetate with the unpleasant TMA. 

We are aware that repeating a test with a different odorant is not only a matter of applying a well consolidated technique. One should also know whether the subjects engaged are already familiar with the odorant. A preliminary test aimed to assess the degree of familiarity is of extreme importance since this parameter strongly affects the measurement of olfaction [47]. To this regard, the “Sniffin’ sticks Screening Test”, was widely used in Europe to check the ability to identify the odor and the degree of familiarity with it. This kind of test was also performed in Italy with normoosmic people, confirming that TMA was recognized within a panel of odorants as a familiar one [48]. Long ago, TMA proved to be the best probe to detect a specific anosmic defect. In Berkeley, this was estimated to occur in around 7% of subjects [26]. In Italy, this value is not known; however, it should be quite easy to identify and discard anosmic subjects from the test on the basis of the Berkeley experience.

In conclusion, it may be interesting to replicate olfactory tests by using trimethylamine instead of amyl acetate. At first, these experiments may offer a valuable alternative to confirm or otherwise the gender-dependent effect on olfaction since the rotten fish odorant is one of the most disgusting, yet familiar, odorants. Secondly, as we have seen, trimethylamine exhibits a strong discriminant significance between male and female humans from a metabolic point of view. Thirdly, the subjects engaged in these olfactory tests could also be interviewed about their dietary habits, in order to evaluate whether there is a significant covariation of olfactory thresholds with fish consumption frequency.

## 3. Covariation between Sex-Dependent Olfaction and Alimentary Habits

A pleasant or an unpleasant food odorant may determine opposite psycho-active effects like attraction or repulsion, respectively. Thus, how a specific odorant affects appetite may be of concern, especially to researchers studying food neurochemistry. In our specific hypothesis, trimethylamine detection seems to be differently tuned according to the hormones in play, so that it may also provide a sex-dependent psychic reaction towards fish as a food. This question is extremely relevant to the ‘total quality’ assessment and definition of food [49]. 

The pivotal role of testosterone, as proposed in Figure 1, may explain at least part of the modulation of olfactory acuity in fertile women. Unfortunately, there are no data yet on olfaction in men. However, testosterone arousal on sexual maturation is normal, so the fact that it may exert a similar effect in men cannot be ruled out.

Among the Italian population, fish consumption has increased in the last decade. A national survey of the Ministry of Health has found that in 2007, 60.14% of consumers ate fish three times per week or more, an overall increase 7.22% since 1997 (see ISTAT, 2008; http://www.istat.it/sanita/Health/). These average values change according to the geographical distribution of the population, with higher consumption in areas where fresh fish is available, such as the islands. In Table 1 we report some values extrapolated from the survey on thousands of male and female subjects. Values range from a minimum of 50.33% in the North East to a maximum in the South (67.27%) and islands (66.29%). Minimal gender differences were noted; however, this data was averaged according to respondents’ age. Although this survey cannot offer statistical validity, it shows how the geographical dishomogeneity of an area may complicate this kind of analysis. 

Among the surveys published by ISTAT, we can collect significant data from 1993 to 2007 (included) on the percentage of Italian women and men, separately, which consume fish at least three times a week. A decline in consumption clearly occurs among the elderly (> 65 years), however, this data was not considered here, since a relation between old age and fish consumption is better tackled by other sources [7]. Instead, we analysed the data relative to 3-14 and 15-44 year-olds respectively, the data were estimated by a two way analysis of variance (ANOVA) with interaction (age and sex as fix effects) and difference of fish consumption between male and female population were compared within each age group based on Student’s t-test. We made our choice because of previous studies involving olfactory tests with subjects of approximately the same ages [5] which meant we could infer whether there was a covariation between fish preferences and olfactory detection thresholds, upon sexual maturation.

**Figure 2:**
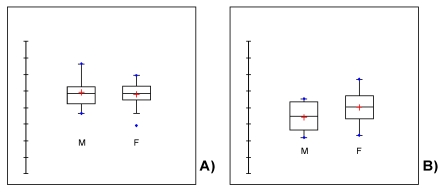
Variation of the percent of Italian consumers who ate fish at least three times a week from 1993 to 2007 (included), for sex and age classes, 3-14 (A) and 15-44 (B) years old.

ANOVA has revealed a highly significant effect of age group (p < 0.001) and a almost significant effect of sex (p = 0.067) and age X sex interaction (p = 0.003). As one can see in Figure 2, there is no difference in fish consumption frequency between female and male pre-pubertal individuals (p = 0.65). However, after sexual maturation, the frequency significantly decreases (p < 0.001) and tends to differentiate according to sex. There is a slightly higher proportion of women who consume fish at least three times per week than fertile men (p < 0.1).

**Table 1:** Percent of Italian women and men consuming fish 3 or more times a week in 2007. Taken from the official statistical database of the Italian Ministry for Health.

Year	Sex	Italy	North-West	North-East	North	Centre	South	Islands
2007	M	59.61	56.05	49.45	53.29	62.26	66.96	64.97
2007	F	60.64	57.00	51.18	54.58	62.16	67.55	67.53
2007	M+F	60.14	56.54	50.33	53.95	62.21	67.27	66.29
Extrapolated from ISTAT (2008). Statistical databases. Available: http://www.istat.it/sanita/Health/								

## 4. Conclusions

The data on fish consumption in Italian women seems to support our hypothesis that sexual maturation may trigger more sensitive olfaction and, thus, a reduction in fish consumption. A significant reduction in fish consumption upon sexual maturation was also observed in Italian men. However, in this case, we cannot draw any conclusions, since a test to verify the possible effect of sexual maturation on olfactory acuity of men is still lacking. 

We have observed a covariation between olfactory “detection” thresholds [5] and fish consumption upon sexual maturation. Whether sexually mature women do consume more fish than men because they are less sensitive to the disgusting odor, has yet to be demonstrated. To this regard, several variables are to be considered. In a brief communication, for instance, it was reported that after repeated tests, a 5-fold amelioration of olfactory acuity towards different odorants could be induced in women in reproductive age but not in men [50]. Moreover, it is known that a repeated exposure to a maleodorant molecule might attenuate the sense of disgust towards this molecule, however we cannot yet predict sex-dependence habituation towards trimethylamine. In other words, how much odor “preference” might differentially affect odor “detection” in males and females must be evaluated with each single odorant. The olfactory data we have used here, refers to tests with the odorant amylacetate, which is why we propose new tests for the future with trimethylamine as the odorant probe. Furthermore, these tests should be carried out in parallel with measurements of the gonadal hormones that might modulate olfactory information processing in central districts beside the olfactory bulb. 

Finally, the ISTAT responses reported here are obtained by a single interview on a random sample and do not consider the monthly chronobiology of the participants and some psychological features. Thus, further research is needed to deepen the understanding of fish consumption patterns and olfactory sensibility and to try to correlate these results with a careful consideration of other psychological factors (e.g. attitudes towards eating fish, health beliefs), that could act as mediators in fish consumption.
